# Caught Between Stewardship and Resistance: How to Treat Acute Complicated Diverticulitis in Areas of Low Antimicrobial Susceptibility?

**DOI:** 10.3390/antibiotics13121150

**Published:** 2024-12-01

**Authors:** Octavian Enciu, Elena-Adelina Toma, Adrian Miron, Gabriela Loredana Popa, Andrei-Alexandru Muntean, Andrei Ludovic Porosnicu, Mircea Ioan Popa

**Affiliations:** 1 Department of Microbiology, “Cantacuzino” Institute, “Carol Davila” University of Medicine and Pharmacy, 020021 Bucharest, Romania; octavian.enciu@umfcd.ro (O.E.); adrian.miron@umfcd.ro (A.M.); dr.gabriela.popa@gmail.com (G.L.P.); alexandru.muntean@umfcd.ro (A.-A.M.); andrei.porosnicu@umfcd.ro (A.L.P.); mircea.ioan.popa@umfcd.ro (M.I.P.); 2 Emergency Hospital-Surgery Department, Elias University, 020021 Bucharest, Romania; 3 Colentina Clinical Hospital-Parasitic Disease Department, 020021 Bucharest, Romania; 4 The “Cantacuzino” National Medico-Military Institute for Research and Development, 020021 Bucharest, Romania

**Keywords:** antimicrobial resistance, acute diverticulitis, multidrug-resistant bacteria, mortality, antibiotic stewardship

## Abstract

Antimicrobial resistance is one of the main threats to public health, with multidrug-resistant (MDR) pathogens on the rise across continents. Although treatment guidelines generally recommend antimicrobial therapy for acute complicated diverticulitis, they do not specify treatment pathways according to local or national resistance profiles. There is sparse data regarding specific pathogens involved in Hinchey II–IV patients who undergo surgery. This study seeks to address these issues and determine how often and what types of MDR bacteria occur in patients undergoing emergency surgery. We prospectively enrolled patients admitted between 2020–2023 and who underwent emergency surgery for complicated acute diverticulitis. We analysed the inflammatory response parameters at admission, the type of surgery employed for source control, identified pathogens in the peritoneal samples, their antimicrobial susceptibility, the efficacy of antimicrobial empiric therapy, and mortality. Gram-negative bacteria were identified most often, with *Escherichia coli* being mostly MDR (43.9%) or extended-spectrum beta-lactamase producing (ESBL +ve) (24.4%), while most strains of *Klebsiella pneumoniae* were extended-spectrum beta-lactamase positive (ESBL +ve) (80%) and MDR (80%). Of the *Enterococcus* spp., 57.14% were vancomycin-resistant (VRE) strains. Patients with Hinchey III/IV were significantly more associated with MDR. Patients with multiple pathogens were significantly associated with ESBL+/VRE strains. Age, leucocytosis, and procalcitonin levels at admission were good indicators for mortality prediction, which occurred in four cases. In an age when antibiotic stewardship is advisable especially in emergency settings, the treatment should be tailored according to local profiles of MDR to ensure adequate outcomes for patients.

## 1. Introduction

### 1.1. Antimicrobial Resistance in Romania

Antimicrobial resistance (AMR) is one of the main concerns of the medical world today, seen customarily as a chronic public health threat that could lead to more than 10 million deaths by 2050, affecting people around the globe, with a particular burden placed on the elderly [1,2]. Drug-resistant pathogens are at the forefront of threats the World Health Organization (WHO) identifies, affecting countries in all regions and income levels. AMR has the potential of reversing many modern health gains, being directly responsible for 1.27 million global deaths in 2019 but contributing to more than 4 million deaths. AMR’s economic cost is enormous, estimated at USD 1 trillion by 2050 by the World Bank [3]. Research priorities to tackle this issue include antibiotic stewardship and drivers of appropriate and inappropriate antimicrobial therapies employed worldwide, a task that can only be accomplished by studying the particularities of local, national, and continental infections in settings with high AMR prevalence and inadequate diagnostic and/or treatment capacities [4]. Moreover, mortality rates in Romania that can be attributed to or associated with drug-resistant pathogens are among the highest in the European Union (EU), with over half of deaths involving infections in 2019 associated with at least one AMR pathogen [5].

Romania places first in the EU and among the top European countries regarding the burden of AMR and deaths attributable to and associated with AMR, with *E. coli* and *K. pneumoniae* being some of the most frequently encountered pathogens displaying various resistance patterns [5]. Romania is implementing a national strategy (2023–2030) to prevent hospital-acquired infections and AMR [6]. The national strategy states that in 2019, Romania had the third highest level of antimicrobial usage, while in veterinary medicine, the usage of antimicrobials was below the European average. Another important issue is the preferential prescription of broad-spectrum antibiotics while the usage of narrow-spectrum antibiotics has decreased. Further measures have been undertaken as the “Matei Balș” National Institute of Infectious Disease was part of a joint project funded by the EU with the Norwegian Institute of Public Health, a country recognised for one of the lowest rates of AMR and strictly controlled antimicrobial usage [7]. The results of this pilot study indicated that doctors should be cautious when prescribing antimicrobial therapy. They suggested that certain antibiotic classes should be included on a list of restricted usage to be used as a last resort, a measure of antibiotic stewardship that has been employed successfully in other countries.

A recent review of drug-resistant ESKAPE pathogens (*Enterococcus faecium*, *Staphylococcus aureus*, *Klebsiella pneumoniae*, *Acinetobacter baumannii*, *Pseudomonas aeruginosa*, and *Enterobacter* spp.) in Romania suggests that the occurrence of AMR in community-acquired infections could be due to antibiotic overuse, as well as the presence of residual antibiotics in the water supply, in nature and the environment, or even in food (particularly in the setting of rising AMR organisms in meat and produce) [8]. However, the study also confirms the worldwide scarcity of data regarding such cases. This confirms the urgency of establishing solid practices regarding antibiotic stewardship while also keeping in mind that optimal therapy might involve newly developed antibiotic classes, which are expensive, sometimes unavailable, and ultimately might perpetuate the issue of MDR.

### 1.2. Therapeutic Options for Acute Complicated Diverticulitis

Colonic diverticulosis is an ailment that affects all sexes and races, with increased prevalence in people over 40 years of age, and can be influenced by genetic factors as well as lifestyle, especially increased body mass index, smoking, and a low-quality diet [9]. Diverticulitis can complicate this disease in up to half of patients with colonic diverticular disease. It may warrant conservative treatment, such as dietary changes, or it may require hospital admission, antibiotic therapy, or surgical management of the disease [10]. The most recent randomised controlled trials (RCTs) and meta-analyses regarding therapeutic options for acute diverticulitis focus on whether to employ antimicrobial therapy at all in the treatment of uncomplicated cases [11,12,13,14] and what the appropriate surgical approach and choice of operation are for patients who must undergo emergency surgery [15]. From a surgical standpoint, the management is currently limited to peritoneal lavage for limited peritoneal contamination in Hinchey II-III diverticulitis or resection of the affected colonic segment. After resection, a terminal stoma can be created (Hartmann’s operation), or an anastomosis can be performed. Depending on availability, the surgeon’s preference and experience, and the patient’s condition, minimally invasive surgery can be employed for the surgical treatment of complicated diverticulitis. Perforation alone is a complication that can lead to death in up to one in five patients, and considering the rising prevalence of diverticulosis, it is an issue for medical caregivers globally [14].

However, glancing across multiple guidelines elaborated by surgeons, gastroenterologists, or multidisciplinary groups, one can observe the absence of specific indications regarding antibiotic regimens in acute complicated diverticulitis, especially in patients who cannot be managed conservatively [16,17,18]. This need has been underlined in the American College of Physicians guideline, which states explicitly that there is inconclusive evidence in published studies regarding antibiotic resistance and there is no evidence addressing antibiotic therapy in patients with complicated acute diverticulitis [19].

The Complicated Intra-Abdominal Infection Observational Study and the Complicated Intra-Abdominal Infection Observational World Group (CIAO and CIAOW) published their results in a 2017 article. They found that of the 363 bacteria isolated in 272 cases, 22 (7%) were drug resistant, 18 (5.7%) of which were community acquired [20]. A multidisciplinary review led by four Italian scientific societies in 2020 regarding the management of perforated diverticulitis stated that while broad-spectrum antimicrobial therapy is indicated, no first line of treatment can be recommended, and surgeons must rely on local profiles, availability, preference, and cost when prescribing the appropriate medication [21]. Studies confirm that *Escherichia coli* is the most frequent organism found in bacterial cultures from peritonitis or intraabdominal abscesses, but data regarding susceptibility to antimicrobials are lacking [22,23].

The present study aimed to identify pathogens and their antimicrobial susceptibility and resistance profiles in community-acquired intraabdominal infections secondary to complicated acute diverticulitis. We also investigated the success or failure of empiric antibiotic therapy and possible risk factors for AMR and multidrug-resistant (MDR) organisms.

## 2. Results

There were 69 patients enrolled in the study, most of them women (55.1%) with an average age of 60.51 ± 16.7 years (median = 62 years). Of the 69 patients, 27 (39.1%) had criteria for septic shock at admission. The mean leucocyte count (white blood count–WBC) was 16,390/μL ± 4680/μL (median = 15,400/μL), the mean C-reactive protein (CRP) was 152.96 ± 107.04 mg/L (median = 113 mg/L), and mean procalcitonin was 5.41 ± 8.49 ng/mL (median = 2.68 ng/mL). Distribution according to the American Association of Anesthesiology (ASA) grading shows that most of the patients were assigned grade II ASA (53.6%), and 42% of the patients had comorbidities, primarily cardiovascular diseases (42%). According to the modified Hinchey classification, most patients had IIB diverticulitis (30 patients—43.5%). Laparoscopic drainage (43.5%) was the most frequent type of surgery used for source control; in 69.6%, there were identified pathogens in the peritoneal fluid or from peritoneal abscesses—75% of the cases involving a single pathogen, and 25% testing positive for multiple concomitant pathogens. The mean Intensive Care Unit (ICU) admission duration was 1.72 ± 1.4 days (median = 1 day), 39.1% of the patients had an ICU stay of more than 1 day, while the mean hospital length of stay (LOS) was 7.87 ± 2.97 days (median = 6 days). Most patients received adequate empiric antibiotherapy (75%), and 5.8% of the patients (four patients) died during admission. There were no other reported deaths during the follow-up period of 30 postoperative days. Table 1 shows the summarised variables of the analysed patients.

Data from Table 2 show the distribution of the patients with identified pathogens according to bacterial type. In most cases, *Escherichia coli* was present alone or in multi-bacterial isolates (85.41%). In cases with single pathogen isolation from culture (36 cases–75%), 32 cases (88.9%) were with *Escherichia coli*, 2 cases were with *Enterococcus faecalis* (5.6%), and 2 cases were with *Klebsiella pneumoniae* (5.6%). In cases with multiple pathogens isolated from cultures (12 patients–25%), the distribution is as follows: six cases with *E. coli* and *K. pneumoniae* (50%), three cases with *E. coli* and *Enterococcus faecium*, one case with *E. faecalis* and *K. pneumoniae*, one case with *E. faecium* and *K. pneumoniae*, and one case with *Veillonella* spp. and *Fusobacterium nucleatum*.

### 2.1. Analysis According to Drug-Resistance Exhibited by Identified Pathogens

Table 3 shows the distribution of the patients with identified pathogens according to bacterial resistance. In the Gram-negative bacteria group, *E. coli* was multidrug resistant in almost half of the cases (43.9%), and 10 strains were ESBL producers (24.4%). Most of the isolated strains of *K. pneumoniae* were ESBL positive (80%) and multidrug resistant (80%). Of the *Enterococcus* spp., 57.14% were VRE.

Correlations between patient characteristics and AMR pathogens are shown in Table A1**.** Most of the analysed parameters were not significantly different depending on the existence of AMR, except for the distribution according to the Hinchey classification (*p* = 0.004) where Z-tests with Bonferroni correction showed that patients with IIB Hinchey diverticulitis were significantly less associated with multidrug resistance (76.5% vs. 25.8%). In comparison, patients with Hinchey III disease were significantly more often associated with multidrug resistance (45.2% vs. 5.9%). Patients with inadequate antibiotherapy were also significantly associated with antimicrobial drug resistance (38.7% vs. 0%) (*p* = 0.004). Moreover, across the study, patients with Hinchey III/IV were significantly more associated with AMR (18 cases–58.1% vs. 2 cases–11.8%), while patients with Hinchey class IIA/IIB were substantially less associated with AMR (15 cases–88.2% vs. 13 cases–41.9%) (*p* = 0.002), having increased odds of resistance by 10.385 times (95% C.I.: 2.017–53.471) (*p* = 0.005).

As stated in Table 4, most patients received broad-spectrum antibiotics at admission, for either single-regimen or combined regimens. More than half of the patients (60%) who received ceftriaxone and metronidazole displayed AMR to third-generation cephalosporins. One-third of patients with MDR pathogens displayed resistance to the fluoroquinolones they were treated with (38.46%), while two patients presented VRE strains resistant to carbapenems.

Multidrug-resistance was also significantly associated with the same parameters, Hinchey class, and inadequate empiric antibiotic therapy, as shown in Table A2. Patients with Hinchey III/IV diverticulitis were significantly more associated with MDR (13 cases–59.1% vs. 7 cases–26.9%), while patients with Hinchey IIA/IIB diverticulitis were substantially less associated with MDR (19 cases–73.1% vs. 9 cases–40.9%) (*p* = 0.039), having increased odds of MDR by 3.921 times (95% C.I.: 1.165–13.198) (*p* = 0.027).

Both risk factors for MDR (Hinchey III/IV and inadequate antibiotherapy), when included in a multivariable binomial logistic regression model, showed independent significance, the inadequate empiric antibiotherapy increasing the odds of MDR by 42.225 times (95% C.I.: 4.002–445.484) (*p* = 0.002), while HINCHEY class III/IV increased the odds of MDR by 7.321 times (95% C.I.: 1.542–34.772) (*p* = 0.012).

Data from Table A3 show the characteristics of the patients with identified pathogens analysed according to the existence of ESBL/VRE. Most parameters were not significantly different according to the existence of ESBL/VRE (*p* > 0.05), except for the following:

- Patients with ESBL/VRE phenotypes had a significantly higher age (66.45 ± 13.58 vs. 55.57 ± 16.96, *p* = 0.022);
- According to Hinchey classification distribution, patients with Hinchey IIB were significantly less associated with ESBL/VRE phenotypes (64.3% vs. 15%), while patients with Hinchey III diverticulitis were significantly more associated with ESBL/VRE pathogens (50% vs. 17.9%) (*p* = 0.002);
- Source control distribution shows a significantly lower frequency of ESBL/VRE in patients with laparoscopic drainage (46.4% vs. 15%), while patients with laparoscopic Hartmann’s procedure were significantly more associated with ESBL/VRE (25% vs. 3.6%) (*p* = 0.018);
- Patients with multiple pathogens isolated in the peritoneal fluid/abscesses were significantly more associated with ESBL/VRE (*p* = 0.016);
- Inadequate empiric antibiotherapy was significantly more associated with ESBL/VRE (50% vs. 7.1%) (*p* = 0.002).

**Figure 1:**
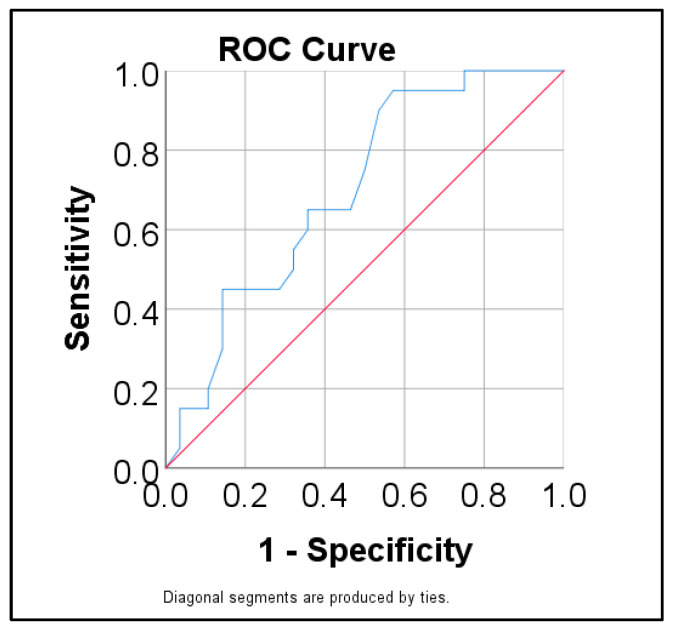
ROC curve for predicting ESBL/VRE pathogens using age.

Data from Table A4 and Figure 1 show the ROC curve for predicting ESBL/VRE using age as a predictor. Age had a significant prediction over ESBL/VRE pathogens (*p* = 0.020) having a moderate performance (AUC = 0.699, 95% C.I.: 0.552–0.846). The cut-off calculated based on the highest Youden value was 50.5, showing that patients with an age of 50.5 years or higher were predicted as having isolates with ESBL/VRE with a 95% sensitivity and 42.9% specificity.

Data from Table 5 show the univariate and multivariate binomial logistic regression models used to predict ESBL/VRE presence in the peritoneal cavity. In univariate models, all parameters had a significant prediction over the existence of ESBL/VRE profiles of resistance (*p* < 0.05). The multivariate model excluded the existence of inadequate antibiotherapy due to multicollinearity. As such, based on the results obtained in the multivariate model, the data show that the following parameters have a significant and independent prediction:

- Hinchey III/IV diverticulitis significantly increases the odds of having ESBL/VRE pathogens by 8.339 times (95% C.I.: 1.72–40.43) (*p* = 0.008);
- The existence of multiple pathogens isolated in the peritoneal fluid or abscesses significantly increases the odds of having ESBL/VRE strains by 7.459 times (95% C.I.: 1.19–46.76) (*p* = 0.032).

### 2.2. Analysis According to Mortality

Out of the 69 patients included in the study, there were four in-hospital deaths, while none of the other patients succumbed during the 30-day follow-up. Data from Table A5 show the characteristics of the patients and how the studied parameters correlated to mortality. Deceased patients were older (83 ± 6.58 vs. 59.12 ± 16.15, *p* = 0.001), and had higher values of WBC (median = 22.19, IQR = 18.27–24.5 vs. median = 15.14, IQR = 13.19–18.21, *p* = 0.013) and procalcitonin at admission (median = 20.93, IQR = 5.08–39.67 vs. median = 2.11, IQR = 0.75–4.9, *p* = 0.009), with longer ICU hospitalisation (median = 3.5, IQR = 2–8 vs. median = 1, IQR = 1–2, *p* = 0.007) and a shorter length of stay (median = 3.5, IQR = 2–8 vs. median = 8, IQR = 6–10, *p* = 0.047) than patients who survived. Patients with septic shock at admission (100% vs. 35.4%, *p* = 0.020) and patients with ASA grade IV (25% vs. 20%) or grade V (75% vs. 0%) (*p* < 0.001) were significantly associated with higher mortality rates. Patients with ASA grade II had better survival rates (56.9% vs. 0%). According to source control distribution, patients who underwent open Hartmann’s procedure died significantly more often (*p* = 0.030).

**Figure 2:**
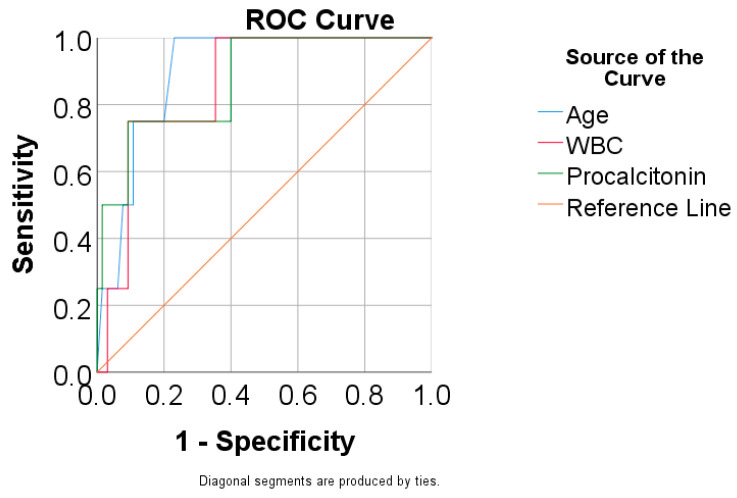
ROC curve for predicting mortality using age, WBC, and procalcitonin.

Table 6 and Figure 2 show the ROC curve for predicting mortality using age, WBC, and procalcitonin. Results show the following:

- Age had a significant and excellent prediction (*p* = 0.008, AUC = 0.900, 95% C.I.: 0.806–0.994), showing that patients with an age of 74 years or higher had increased predictive mortality rates, with 100% sensitivity and 76.9% specificity;
- WBC had a significant and very good prediction (*p* = 0.017, AUC = 0.858, 95% C.I.: 0.719–0.996), showing that patients with a value for WBC of 21,570/μL or higher had increased mortality rates, with 75% sensitivity and 90.8% specificity;
- Procalcitonin had a significant and very good prediction (*p* = 0.013, AUC = 0.873, 95% C.I.: 0.707–1.000), showing that patients with a value for procalcitonin of 10,68 or higher were predicted as having increased mortality rates with 75% sensitivity and 90.8% specificity.

Univariate and multivariate binomial logistic regression models used in the prediction of mortality using analysed parameters are shown in Table A6. In univariate models, all parameters (age, WBC, procalcitonin levels, and ICU admission) had a significant prediction over mortality (*p* < 0.05). In the multivariate model, all variables lacked significant prediction (*p* > 0.05), probably due to the small number of deaths (*n* = 4).

As presented in Table A7, an alternative multivariable model was constructed using the stepwise forward method, which selected WBC and ICU duration as significant predictors. This model showed the following:

- Each additional 1000 leukocytes/μL increased the odds of death by 1244 times (95% C.I.: 1011–1532);
- Each increase by one day in ICU admission increased the odds of death by 2153 times (95% C.I.: 1220–3797).

## 3. Discussion

The results of this study indicate a high prevalence of drug-resistant community-acquired bacteria involved in peritonitis secondary to acute complicated diverticulitis, with age, Hinchey III/IV class, pluri-bacterial infections, and inadequate antibiotherapy as significant predictors of such cases. Mortality is also correlated with age, leukocytosis, sepsis, and longer ICU admission. The results of our study are, however, slightly better in terms of mortality than those reported in other studies [20,24]. Empiric antibiotic therapy is identified in most studies as necessary to obtain good outcomes. Still, guidelines and extensive reviews either lack specific recommendations, have not been updated in more than a decade, or do not apply to areas with high AMR [25,26]. This has been recognised as an important issue in the literature, with scarce evidence regarding antimicrobial therapy in acute colonic diverticulitis, which forces doctors to rely on personal preference, tradition, and indirect evidence when prescribing empiric antibiotic treatment [27,28,29,30].

Risk management strategies in the treatment of acute complicated diverticulitis in an emergency setting should involve factors such as multidisciplinary teams, analysis of individual factors at admission (such as age, comorbidities, risk factors for hospital-acquired infections), signs of sepsis, and adequate source control for patients in need of surgical intervention [31]. This study enrolled patients starting in 2020 when the COVID-19 pandemic was in full force worldwide. This affected the management options for surgeons in the beginning, as national and international professional societies struggled to find the appropriate timing of surgery and debated the safety of laparoscopic surgery for emergencies [32,33]. However, as the SARS-CoV-2 crisis subsided, the question of laparoscopic drainage, colonic resection and anastomosis, or Hartmann’s procedure still lingers when it comes to the appropriate management of complicated diverticulitis that cannot be treated conservatively. Still, data suggest that primary digestive resection and anastomosis are equally successful or even better than the previously-preferred resection and end colostomy [34,35,36]. The results presented in our study confirmed this, as the surgical technique employed did not correlate with AMR. Still, Hartmann’s procedure was associated with higher mortality rates—an expected outcome, as this remains the first option for high-risk patients who are in critical condition [37].

Leukocytosis is one of the most widely looked at clinical factors in deciding whether patients who present to the ER with abdominal pain should be admitted and undergo surgical treatment. However, in acute diverticulitis, some studies show that WBC cannot be used as a predictor in evaluating disease severity and does not correlate with mortality [38,39]. Our study did find that an increase of 1000 cells/μL increased the odds of death by 1.244, and high WBC at admission did correlate with mortality, but no cut-off value could be indicated. In contrast, high C-reactive protein values have been repeatedly identified as good predictors for complicated diverticulitis with more severe outcomes. At the same time, a rise in CRP values after initial conservative management and persistently elevated results after ICU admission have also been good indicators of high morbidity and mortality [40]. The threshold identified by most studies was a value of CRP greater than 150 mg/L (in some articles greater than 175 or 200) in predicting the risk of perforation or other complications, failure of conservative treatment, likelihood of urgent surgery, and mortality [39,40,41,42]. The studied lot did not yield significant correlations regarding CRP values regarding AMR risk or mortality. Still, all patients had elevated levels at admission, as expected, considering all patients underwent emergency surgery. We found that procalcitonin was also a good predictor of mortality but had no significant bearing on AMR. Other authors have suggested that a PCT value of ≥ 10 ng/mL strongly predicted short-term mortality, with even better prediction values correlated with lactate levels [43,44].

In our study, ICU length of stay of more than one day was strongly associated with higher mortality rates, and every additional day in the ICU increased the odds of death by 2.153 times. Length of intensive care admission has been linked to higher mortality rates in other studies as well, across multiple countries and determinants of disease, which is to be expected, as advanced life support measures taken in such settings are indicators of severe disease in most patients [45].

Most patients we included in the study had *E. coli* identified in the peritoneal cultures, either as a single pathogen or in multibacterial infections, followed by *Klebsiella* spp. and *Enterococcus* spp., which is in line with other intraabdominal infection isolates described in the literature in patients with acute diverticulitis [20,22]. Unlike previous reports, we did not find any fungal species nor any of the frequent anaerobes we expected. However, we encountered one case that was associated with *Veillonella* spp. and *Fusobacterium nucleatum*, which are usually commensal microbes present in the oral cavity. *Fusobacterium* spp. has emerged in the last decade as a new pathogen involved in osteomyelitis, liver and lung abscesses, appendicitis, and bacteraemia [46,47]. Some authors have pointed to preexisting diverticulosis of the colon with recurrent diverticulitis as a possible source, most commonly in immunocompromised patients [48]. The rates of AMR (be it resistance to one antibiotic class, MDR, or ESBL/VRE strains) were higher in our group than those reported by Coccolini et al. for *Klebsiella* spp. and *Enterococcus* spp. Still, the analysis yielded similar results for *E. coli* isolates [20]. Failure of empiric antibiotic therapy was identified in both our study and the aforementioned one as a risk factor for AMR, confirming the need to adapt guidelines to local microbiological profiles and enforce guided antibiotic stewardship.

## 4. Materials and Methods

### 4.1. Study Design, Population and Eligibility Criteria

This prospective cohort observational study was conducted between the 1st of October 2020 and 31 December 2023, in the Surgery Department of Elias Emergency Hospital, Bucharest, Romania. We evaluated all patients admitted in an emergency setting during this period. We enrolled those who fit the subsequent criteria: intraoperative diagnosis of acute complicated diverticulitis (Hinchey modified classification II–IV), who underwent emergency surgery during the first 24 h following admission, who did not meet any criteria for exposure to healthcare-associated infections according to the definition proposed by Friedman et al. in 2002 [49].

### 4.2. Data Collection

The variables included in the study were demographic information, preexisting comorbidities, clinical condition at admission (and whether patients fulfilled the criteria for sepsis or septic shock [50]), blood test results (with a focus on white blood count, C-reactive protein and procalcitonin), American Society of Anesthesiology (ASA) grade, source control employed (surgical technique), modified Hinchey class (according to Sher et al. [51]). We also registered the results of bacteriological tests from the peritoneal liquid or abscess retrieved during surgery, duration of admission to the Intensive Care Unit (ICU), empiric antibiotherapy employed, and length of hospital stay (LOS).

### 4.3. Microbiological Analysis and Antimicrobial Susceptibility Testing

To appraise antimicrobial susceptibility, classical bacteriology methods were employed to identify bacterial isolates, with serial dilution and filtration of samples and cultivation on solid agar-based media (blood agar, chocolate agar, and MacConkey agar). Afterward, the inoculated media were stored for 48 h to allow incubation at 37 °C, followed by further analysis of the obtained colonies through biochemical testing, using multitesting and automated testing. Subsequent disk diffusion was used according to the Kirby-Bauer method, as well as broth microdilution on automated biochemical analysers Vitek 2 (bioMérieux, Marcy-l’Etoile, France) and BD Phoenix (Becton Dickinson, Oxford, UK). ESBL (extended-spectrum beta-lactamase)-producing strains were identified using the double diffusion test on the same automatic analysers. Minimum inhibitory concentration (MIC) was used to indicate antibiotic sensibility, and the protocol to determine MIC, as well as consequential results were interpreted according to the European Committee on Antimicrobial Susceptibility Testing (EUCAST) guidelines [52]. Gram-positive isolates were tested to determine MIC using the following antibiotic agents: ampicillin, gentamycin, vancomycin, teicoplanin, linezolid, and tigecyline, while for Gram-negative bacteria, the following were used: ampicillin, amoxicillin/clavulanic acid, piperacillin/tazobactam, ceftriaxone, ceftazidime, cefotaxime, aztreonam, ertapenem, meropenem, imipenem, gentamycin, amikacin, trimethoprim/sulfamethoxazole, levofloxacin, and colistin. For strains that exhibited resistance patterns, additional testing was performed to determine MIC for ceftolozane/tazobactam, ceftazidime/avibactam, meropenem/vaborbactam, and imipenem/relebactam. Susceptibility was defined per EUCAST standards as a likely therapeutic success to standard dosing regimens, while also considering those strains that exhibited susceptibility at increased exposure rates as non-resistant. Drug resistance was defined as bacteria being non-susceptible to at least one agent from one antibiotic class, even with increased exposure by adjusting the dosing regimen. In comparison, MDR was defined as being non-susceptible to at least one agent from three or more classes [52,53].

### 4.4. Statistical Analysis

The primary outcomes we reviewed were the types of pathogens displaying AMR and mortality (during hospital stay and the 30-day immediate follow-up).

All the data from the study were analysed using IBM SPSS Statistics 25 and illustrated using Microsoft Office Excel/Word 2021. Quantitative variables were tested for normal distribution using the Shapiro–Wilk Test and were written as averages with standard deviations or medians with interquartile ranges. Quantitative variables with non-parametric distribution were tested between groups using the Mann–Whitney U Test. Quantitative variables with normal distribution were tested between groups using Student’s T-Test/Welch T-Test (based on the equality of variances between groups observed by the Levene test). Qualitative variables were written as counts or percentages and were tested between groups using Fisher’s Exact Test. Z-tests with Bonferroni correction were used to further detail the results obtained in the contingency tables. ROC (receiver operating characteristic) curves were used to analyse prediction performance over important factors based on different quantitative variables. The performance was quantified using AUC values with 95% confidence intervals and significance values. Cut-off values were selected based on the highest Youden index value, and the cut-off values’ prediction performance was measured using sensitivity and specificity. Univariable and multivariable logistic regression models were used to predict important factors (bacterial resistance and mortality); the measure of prediction was estimated using odds ratios with 95% confidence intervals and significance values. Models were tested for significance and goodness of fit.

The threshold for the significance level for all tests was α = 0.05.

## 5. Conclusions

Antibiotic stewardship is becoming a global necessity due to the challenges posed by rising antimicrobial resistance. When faced with surgical emergencies such as complicated acute diverticulitis that cannot be managed conservatively, treatment should be tailored according to local profiles of MDR to ensure adequate outcomes for patients.

Our study suggests that patients over the age of 50.5, those with Hinchey III/IV diverticulitis, and those with multiple pathogens isolated in the peritoneal fluid had higher chances of an MDR infection. Mortality was associated with age, WBC, procalcitonin levels, and ICU admission.

## 6. Limitations

This study was conducted during the height of the COVID-19 pandemic, which made it difficult to conform to the usual guidelines regarding all medical and surgical treatments in place beforehand. The number of enrolled patients is small, but the only other study that addressed this issue had four times as many patients from multiple centres—further studies on larger populations are needed to make tailored recommendations for patients suffering from complicated diverticulitis. Selection bias was also related to unreported differences in patients—comorbidities such as smoking, obesity, or previous COVID-19 infection were not included in the analysis, outside of the defined criteria for hospital-acquired infections. Medical information about the patients’ medical histories and drug therapies could have been inaccurate, especially for older patients who lived alone and did not have much contact with family members due to restrictions in place at the time of hospital admission. Moreover, patients underwent surgery performed by multiple surgical teams with different degrees of experience and, at times, antimicrobial availability was limited due to the COVID-19 pandemic and disruptions in the supply chains worldwide.

## Figures and Tables

**Table 1:** Patient variables.

Parameter (*n* = 69 Patients)	Value
Gender (male) (Nr., %)	31 (44.9%)
Age (mean ± SD, median (IQR))	60.51 ± 16.7, 62 (49.5–75)
Septic shock (Nr., %)	27 (39.1%)
WBC (mean ± SD, median (IQR))	16,390 ± 4680, 15.4 (13,411–18,560)
CRP (mean ± SD, median (IQR))	152.96 ± 107.04, 113 (82.5–214)
Procalcitonin (mean ± SD, median (IQR))	5.41 ± 8.49, 2.68 (0.8–5.4)
ASA Grading (Nr., %)	
I	2 (2.9%)
II	37 (53.6%)
III	13 (18.8%)
IV	14 (20.3%)
V	3 (4.3%)
*Comorbidities* (Nr., %)	29 (42%)
Cardiovascular diseases (Nr., %)	29 (42%)
Diabetes mellitus type II (Nr., %)	3 (4.3%)
*Modified Hinchey classification* (Nr., %)	
IIA	17 (24.6%)
IIB	30 (43.5%)
III	15 (21.7%)
IV	7 (10.1%)
*Source control* (Nr., %)	
Laparoscopic drainage	30 (43.5%)
Laparoscopic resection + anastomosis	12 (17.4%)
Laparoscopic Hartmann	6 (8.7%)
Open resection + anastomosis	3 (4.3%)
Open Hartmann	18 (26.1%)
Identified pathogens (Nr., %)	48 (69.6%)
*ICU admission (mean ± SD, median (IQR))*	1.72 ± 1.4, 1 (1–2)
ICU admission > 1 day (Nr., %)	27 (39.1%)
LOS (mean ± SD, median (IQR))	7.87 ± 2.97, 8 (6–10)
LOS < 7 days	25 (36.2%)
LOS 7–10 days	31 (44.9%)
LOS > 10 days	13 (18.8%)
Empiric antibiotherapy–adequate (Nr., %)	36 (75%)
Mortality (Nr., %)	4 (5.8%)

**Table 2:** Distribution of identified pathogens according to bacterial type.

Bacteria	Value (Nr., %) *n* = 48
**Gram negative**	
*Escherichia coli*	41 (85.41%)
*Klebsiella pneumoniae*	10 (20.83%)
Other (*Veillonella* spp., *Fusobacterium nucleatum*)	1 (2%)
**Gram positive**	
*Enterococcus faecalis*	3 (6.25%)
*Enterococcus faecium*	4 (8.33%)

**Table 3:** Distribution of patients with identified pathogens according to bacterial resistance.

Resistance Profile	Value (Nr., % of Total)
**Gram negative**	
*Escherichia coli*–1 class AB resistance	5 (12.2%)
*Escherichia coli*–ESBL +ve	10 (24.4%)
*Escherichia coli*–MDR (including ESBL +ve)	18 (43.9%)
*Klebsiella pneumoniae*–ESBL +ve/MDR	8 (80%)
**Gram positive**	
*Enterococcus* VRE	4 (57.14%)

**Table 4:** Inadequacy of empiric antibiotic therapy.

Antibiotic Treatment	Value (Nr., % of Total)
Amoxicillin/clavulanate	2 (100%)
Ceftriaxone + Metronidazole	3 (60%)
Cefuroxime + Metronidazole	0
Ciprofloxacin + Metronidazole	5 (38.46%)
Tigecycline	0
Ertapenem	1 (6.25%)
Meropenem	1 (4.17%)
Meropenem + Vancomycin	0

**Table 5:** Univariate and multivariate binomial logistic regression models used in the prediction of ESBL/VRE resistance using analysed parameters.

Parameter	Univariable		Multivariable	
	OR (95% C.I.)	*p*	OR (95% C.I.)	*p*
**Age ≥ 50.5 years**	14.25 (1.667–121.8)	**0.015**	6.659 (0.676–65.58)	0.104
**Hinchey class III/IV**	8.556 (2.297–31.871)	**0.001**	8.339 (1.72–40.43)	**0.008**
**Multiple pathogens**	6.818 (1.542–30.152)	**0.011**	7.459 (1.19–46.76)	**0.032**
**Inadequate AB therapy**	13 (2.413–70.051)	**0.003**	-	-

**Table 6:** ROC curve for predicting mortality using age, WBC, and procalcitonin.

Parameter	AUC (95% C.I.)	Std. Error	*p*	Cut-Off	*Se*	*Sp*
**Age**	0.900 (0.806–0.994)	0.048	**0.008**	74	100%	76.9%
**WBC**	0.858 (0.719–0.996)	0.071	**0.017**	21,570	75%	90.8%
**Procalcitonin**	0.873 (0.707–1.000)	0.085	**0.013**	10.68	75%	90.8%

## Data Availability

For ethical and confidentiality reasons, the data presented in this study are available upon request from the corresponding author.

## Appendix A

**Table A1:** Characteristics of the patients with identified pathogens analysed according to AMR.

AMR	Value (Nr., %/Mean ± SD/Median (IQR))		*p*
	Absent (*n* = 17)	Present (*n* = 31)	
Age	55.71 ± 16.67	62.52 ± 16.02	0.172 *
Gender (male)	7 (41.2%)	14 (45.2%)	1.000 **
Septic shock	5 (29.4%)	11 (35.5%)	0.757 **
WBC	14,560 (12,060–18,370)	15,650 (14,210–19,540)	0.456 ***
CRP	98 (66–190.5)	116 (82–211)	0.264 ***
Procalcitonin	2.73 (0.54–6.34)	3.12 (1.3–9.18)	0.301 ***
*ASA grading*			
I	1 (5.9%)	1 (3.2%)	0.360 **
II	12 (70.6%)	14 (45.2%)	
III	3 (17.6%)	7 (22.6%)	
IV	1 (5.9%)	6 (19.4%)	
V	0 (0%)	3 (9.7%)	
*Comorbidities*	6 (35.3%)	16 (51.6%)	0.368 **
Cardiovascular disease	6 (35.3%)	16 (51.6%)	0.368 **
Diabetes mellitus	0 (0%)	1 (3.2%)	1.000 **
*Hinchey class*			
IIA	2 (11.8%)	5 (16.1%)	**0.004** **
IIB	13 (76.5%)	8 (25.8%)	
III	1 (5.9%)	14 (45.2%)	
IV	1 (5.9%)	4 (12.9%)	
*Source control procedure*			
Laparoscopic drainage	7 (41.2%)	9 (29%)	0.159 **
Laparoscopic resection + anastomosis	4 (23.5%)	5 (16.1%)	
Laparoscopic Hartmann’s procedure	1 (5.9%)	5 (16.1%)	
Open resection + anastomosis	2 (11.8%)	0 (0%)	
Open Hartmann’s procedure	3 (17.6%)	12 (38.7%)	
Multiple pathogens	3 (17.6%)	9 (29%)	0.497 **
ICU admission > 1 day	5 (29.4%)	14 (45.2%)	0.363 **
*Length of stay*			
<7 days	6 (35.3%)	9 (29%)	0.273 **
7–10 days	10 (58.8%)	14 (45.2%)	
>10 days	1 (5.9%)	8 (25.8%)	
Adequate empiric AB therapy	17 (100%)	19 (61.3%)	**0.004** **
Mortality	0 (0%)	3 (9.7%)	0.543 **

* Student T-Test, ** Fisher’s Exact Test, *** Mann–Whitney U Test.

**Table A2:** Patients with identified pathogens analysed according to the existence of MDR.

MDR	Value (Nr., %/Mean ± SD/Median (IQR))		*p*
	Absent (*n* = 26)	Present (*n* = 22)	
Age	57.35 ± 16.34	63.36 ± 16.26	0.209 *
Gender (male)	13 (50%)	8 (36.4%)	0.393 **
Septic shock	7 (26.9%)	9 (40.9%)	0.366 **
WBC	15,400 (13,230–18,290)	15,640 (13,630–19,940)	0.456 ***
CRP	93.5 (76–176.75)	135.5 (86.75–215.5)	0.264 ***
Procalcitonin	2.67 (0.75–6.32)	3.28 (1.76–9.23)	0.301 ***
*ASA grading*			
I	1 (3.8%)	1 (4.5%)	0.170 **
II	18 (69.2%)	8 (36.4%)	
III	3 (11.5%)	7 (31.8%)	
IV	3 (11.5%)	4 (18.2%)	
V	1 (3.8%)	2 (9.1%)	
Comorbidities	10 (38.5%)	12 (54.5%)	0.384 **
Cardiovascular disease	10 (38.5%)	12 (54.5%)	0.384 **
Diabetes mellitus	0 (0%)	1 (4.5%)	0.458 **
*Hinchey class*			
IIA	3 (11.5%)	4 (18.2%)	**0.042** **
IIB	**16 (61.5%)**	**5 (22.7%)**	
III	6 (23.1%)	9 (40.9%)	
IV	1 (3.8%)	4 (18.2%)	
*Source control*			
Laparoscopic drainage	11 (42.3%)	5 (22.7%)	0.157 **
Laparoscopic resection + anastomosis	5 (19.2%)	4 (18.2%)	
Laparoscopic Hartmann’s procedure	1 (3.8%)	5 (22.7%)	
Open resection + anastomosis	2 (7.7%)	0 (0%)	
Open Hartmann’s procedure	7 (26.9%)	8 (36.4%)	
Multiple pathogens	4 (15.4%)	8 (36.4%)	0.111 **
ICU admission > 1 day	8 (3.8%)	11 (50%)	0.239 **
*Length of stay*			
<7 days	8 (30.8%)	7 (31.8%)	0.348 **
7–10 days	15 (57.7%)	9 (40.9%)	
>10 days	3 (11.5%)	6 (27.3%)	
Adequate empiric AB therapy	25 (96.2%)	11 (50%)	**<0.001** **
Mortality	1 (3.8%)	2 (9.1%)	0.587 **

* Student T-Test, ** Fisher’s Exact Test, *** Mann–Whitney U Test.

**Table A3:** Characteristics of the patients with identified pathogens analysed according to the existence of ESBL/VRE.

ESBL/VRE	Value (Nr., %/Mean ± SD/Median (IQR))		*p*
	Absent (*n* = 28)	Present (*n* = 20)	
Age	55.57 ± 16.96	66.45 ± 13.58	**0.022** *
Gender (male)	15 (53.6%)	6 (30%)	0.144 **
Septic shock	7 (25%)	9 (45%)	0.216 **
WBC	15,060 (13,160–18,140)	16,320 (1380–21,770)	0.171 ***
CRP	99 (78.25–165)	153 (84.25–259.75)	0.164 ***
Procalcitonin	2.32 (0.79–6.24)	3.37 (1.68–10.09)	0.250 ***
*ASA grading*			
I	2 (7.1%)	0 (0%)	0.078 **
II	18 (64.3%)	8 (40%)	
III	4 (14.3%)	6 (30%)	
IV	4 (14.3%)	3 (15%)	
V	0 (0%)	3 (15%)	
Comorbidities	10 (35.7%)	12 (60%)	0.143 **
Cardiovascular disease	10 (35.7%)	12 (60%)	0.143 **
Diabetes mellitus	0 (0%)	1 (5%)	0.417 **
*Hinchey class*			
IIA	4 (14.3%)	3 (15%)	**0.002** **
IIB	**18 (64.3%)**	**3 (15%)**	
III	**5 (17.9%)**	**10 (50%)**	
IV	1 (3.6%)	4 (20%)	
*Source control*			
Laparoscopic drainage	**13 (46.4%)**	**3 (15%)**	**0.018** **
Laparoscopic resection + anastomosis	6 (21.4%)	3 (15%)	
Laparoscopic Hartmann’s procedure	**1 (3.6%)**	**5 (25%)**	
Open resection + anastomosis	2 (7.1%)	0 (0%)	
Open Hartmann’s procedure	6 (21.4%)	9 (45%)	
Multiple pathogens	3 (10.7%)	9 (45%)	**0.016** **
ICU admission > 1 day	9 (12.1%)	10 (50%)	0.245 **
*Length of stay*			
<7 days	9 (32.1%)	6 (30%)	0.680 **
7–10 days	15 (53.6%)	9 (45%)	
>10 days	4 (14.3%)	5 (25%)	
Adequate empiric AB therapy	26 (92.9%)	10 (50%)	**0.002** **
Mortality	0 (0%)	3 (15%)	0.066 **

* Student T-Test, ** Fisher’s Exact Test, *** Mann–Whitney U Test.

**Table A4:** ROC curve for predicting ESBL/VRE resistance using age.

Parameter	AUC (95% C.I.)	Std. Error	*p*	Cut-Off	*Se*	*Sp*
**Age**	0.699 (0.552–0.846)	0.075	**0.020**	50.5	95%	42.9%

**Table A5:** Characteristics of the patients according to mortality.

Mortality	Value (Nr., %/Mean ± SD/Median (IQR))		*p*
	Survivors (*n* = 65)	Deceased (*n* = 4)	
**Age**	59.12 ± 16.15	83 ± 6.58	**0.001** *
**Gender (male)**	31 (47.7%)	0 (0%)	0.122 **
**Septic shock**	23 (35.4%)	4 (100%)	**0.020** **
**WBC**	15.14 (13.19–18.21)	22.19 (18.27–24.5)	**0.013** ***
**CRP**	112 (82–211.5)	226.5 (162.5–322)	0.057 ***
**Procalcitonin**	2.11 (0.75–4.9)	20.93 (5.08–39.67)	**0.009** ***
***ASA grading***			
**I**	2 (3.1%)	0 (0%)	**<0.001** **
**II**	**37 (56.9%)**	**0 (0%)**	
**III**	13 (20%)	0 (0%)	
**IV**	**13 (20%)**	**1 (25%)**	
**V**	**0 (0%)**	**3 (75%)**	
**Comorbidities**	26 (40%)	3 (75%)	0.302 **
Cardiovascular disease	26 (40%)	3 (75%)	0.302 **
Diabetes mellitus	2 (3.1%)	1 (25%)	0.166 **
***Hinchey class***			
IIA	17 (26.2%)	0 (0%)	**0.007** **
IIB	30 (46.2%)	0 (0%)	
III	13 (20%)	2 (50%)	
IV	**5 (7.7%)**	**2 (50%)**	
***Source control***			
Laparoscopic drainage	30 (46.2%)	0 (0%)	0.030 **
Laparoscopic resection + anastomosis	12 (18.5%)	0 (0%)	
Laparoscopic Hartmann’s procedure	6 (9.2%)	0 (0%)	
Open resection + anastomosis	3 (4.6%)	0 (0%)	
Open Hartmann’s procedure	**14 (21.5%)**	**4 (100%)**	
**Multiple pathogens**	10 (22.2%)	2 (66.7%)	0.150 **
**ICU admission (days)**	1 (1–2)	3.5 (2–8)	**0.007 *****
**Length of stay (days)**	8 (6–10)	3.5 (2–8)	**0.047 *****
**Adequate empiric AB therapy**	34 (75.6%)	2 (66.7%)	1.000 **

* Welch T-Test, ** Fisher’s Exact Test, *** Mann–Whitney U Test.

**Table A6:** Univariate and multivariate binomial logistic regression models used in the prediction of mortality using analysed parameters.

Parameter	Univariable		Multivariable	
	OR (95% C.I.)	*p*	OR (95% C.I.)	*p*
Age	1.179 (1.011–1.376)	**0.036**	1.210 (0.943–1.551)	0.134
WBC	1.201 (1.004–1.437)	**0.045**	1.203 (0.915–1.581)	0.186
Procalcitonin	1.123 (1.036–1.218)	**0.005**	1.047 (0.905–1.212)	0.533
ICU admission	2.013 (1.195–3.392)	**0.009**	1.603 (0.630–4.078)	0.322

**Table A7:** Multivariate binomial logistic regression model constructed using the step-wise forward (Wald) method used in the prediction of mortality.

Parameter	OR (95% C.I.)	*p*
WBC	1.244 (1.011–1.532)	**0.040**
ICU admission	2.152 (1.220–3.797)	**0.008**
